# Qualitative study exploring knowledge and attitudes towards dementia risk prediction, barriers to dementia services and service improvement recommendations with diverse populations in England

**DOI:** 10.1136/bmjopen-2024-092370

**Published:** 2025-05-30

**Authors:** Rupinder Bajwa, Matilda Hanjari, Amani Al-Oraibi, Ralph Akyea, Manjot Brar, Louise Robinson, Blossom C M Stephan, Nadeem Qureshi, Manpreet Bains

**Affiliations:** 1 University of Nottingham, Nottingham, UK; 2 Newcastle University, Newcastle upon Tyne, UK; 3 Curtin University, Perth, Western Australia, Australia

**Keywords:** Dementia, QUALITATIVE RESEARCH, Health Equity, Health Services, Risk Factors

## Abstract

**Abstract:**

**Objectives:**

This study explored knowledge of dementia, attitudes towards dementia risk prediction and barriers and facilitators to accessing dementia services for diverse populations in England.

**Design:**

Qualitative study using task group methodology, interrogated through framework analysis.

**Setting:**

Task groups were held primarily in-person at local community venues (n=12) with one task group conducted online.

**Participants:**

147 individuals (mean age=63 years old, 62% female) were recruited, representing low-income and ethnically diverse groups from two sites (Nottingham and Newcastle, UK). Participants were from diverse ethnic backgrounds with 37% Black or Black British, 24% Asian or Asian British, 20% white, 9% not provided, 7% Arab and 1% other ethnicities.

**Results:**

Participants possessed some knowledge about dementia but highlighted a need for better access to information about dementia. Participants were knowledgeable about dementia risk factors, but knowledge of risk prediction was low. Attitudes towards dementia risk prediction were cautiously optimistic, and the use of risk prediction tools was viewed as empowering. However, participants stressed the need to consider the psychological impact of a high-risk result. Barriers to accessing dementia services included stigma, denial, language, cultural and religious views about dementia. Recommendations for service improvement included engaging with communities in their spaces, workforce training around dementia awareness, cultural competency and communicating with diverse groups, improving the provision of information in different languages and access to translators.

**Conclusions:**

As international policy on dementia shifts focus to prevention, there is a growing interest in identifying those at high risk and intervening early. This study illustrates current levels of dementia knowledge and attitudes towards risk prediction among socioeconomically and ethnically diverse groups in the UK. Barriers to health services for diverse populations and service improvement recommendations offer a starting point for providers to develop culturally aware and inclusive dementia services.

**STRENGTHS AND LIMITATIONS OF THIS STUDY**

- We recruited a large sample of participants from low-income and culturally diverse communities in England.
- We engaged in meaningful and reciprocal community engagement practices to enhance researcher–community relationships.
- We did not recruit participants from other ethnic minority groups that experience notable inequalities (eg, Gypsy, Roma, Traveller and Bangladeshi communities).

## Background

 Evidence shows there are at least 14 modifiable risk factors for dementia accounting for 45% of cases worldwide.^1^ With the absence of a cure and the number of cases estimated to triple by 2050, there is a critical need to identify individuals with a higher risk of dementia via prediction modelling.^2 3^ Despite this, systematic reviews show that current models tend to have low predictive accuracy, often poor external validity and are not recommended for predicting dementia risk in clinical settings in the UK, or anywhere else globally.^1^,^3^ This is in stark contrast to other public health issues, such as cardiovascular disease, where risk prediction tools are routinely used by health professionals in primary care, to provide disease-risk communication and preventative advice to patients.^4^

Most available dementia prediction models have been developed using data from affluent Caucasian populations.^5^ The reliability of these models has been scrutinised in general. Unsurprisingly then, the predictive utility of these models among underserved groups is also problematic and of concern. Underserved groups are at higher risk for dementia, with studies demonstrating a link between both individual-level and area-level socioeconomic deprivation and higher dementia risk.^6 7^ Ethnic minority groups are also at increased risk of dementia, with individuals from black and South Asian backgrounds being at a greater risk than white individuals.^8^ The intersection between lower socioeconomic status (SES) and belonging to an ethnic minority group may heighten dementia risk further.^9 10^ Evidence suggests low SES and ethnic minority groups already face health inequalities in the context of early diagnosis and access to treatment,^11 12^ which could be exacerbated further if screening and risk prediction tools continue to fall short. Development of risk assessment tools is just one component though; there is limited engagement with people representing both low SES and/or diverse ethnic groups, to understand their views on dementia risk screening. For instance, the transferability of findings from research based on affluent Caucasian populations, which shows these groups are interested in dementia risk assessment, provided measures were objective and deliverable during a routine health check-up,^13 14^ is questionable. Moreover, another systematic review lacking diversity across the included studies concluded that dementia screening (a method used to identify dementia risk) is complex and not likely to be acceptable to the public.^15^

A recent update of the Martin *et al*^15^ systematic review focusing on the concerns of low-income and ethnic minority groups found the latter were willing to undergo dementia screening.^16^ Predictors of willingness included a belief in the benefits of earlier detection (earlier access to treatment) and a desire to boost diversity in research. Unwillingness was related to the psychological implications of a high-risk result.^16^ Two of the reviewed studies noted practical considerations for ethnic minority groups including language barriers, transportation issues and a lack of confidence in medical care.^16^ The only study examining socioeconomic differences reported that lower socioeconomic groups were more sceptical of dementia screening than middle and high socioeconomic groups.^16^ Considering the paucity of studies in this review and the culturally and economically specific perceptions it revealed, there is a need to understand further the views of low-income and ethnic minority groups about dementia risk assessment. Also, with sharp rises in dementia predicted among low SES and ethnic minority groups,^17^ and limited research available with these groups on perceptions of dementia, dementia risk prediction and accessibility of dementia services,^18^ it is necessary to engage with these communities to explore their views further and inform inclusive development of dementia risk prediction tools and services.

### Aims

Engage with underserved groups to explore knowledge of dementia, related risk factors and dementia risk assessment (including barriers and facilitators to implementation) and to collate views and experiences on the accessibility of dementia services.

## Methods

### Design

A constructionist approach underpinned the research. Participants’ words were given meaning and interpreted through the context of their social, cultural and wider environments.^19^ The research team comprised early, mid-career and senior researchers from diverse genders, ethnicities, educational and professional backgrounds with varying migration statuses. The researchers’ professional backgrounds included medicine, pharmacy, education, public health, social work and health policy, and they are experienced in qualitative research methods. Researchers were reflexive in their approach and employed strategies such as bracketing to identify and mitigate prior assumptions.^20^ The COREQ (consolidated criteria for reporting qualitative research) framework supported transparent reporting (see online supplemental material 1).^21^

### Study setting, participants and recruitment

Individuals were purposefully sampled to represent three groups: (1) low-income, (2) South Asian, (3) African-Caribbean, across Newcastle and Nottingham, England. The sampling frame was derived to ensure inclusion of various higher dementia risk profiles, lived experience of dementia (including carers), differing SES and ethnicity, both sexes and those aged 40–74 years. Our previous work showed individuals would prefer that dementia risk assessment be aligned with other health checks,^13 14^ and 40–74 years (inclusive) is consistent with the invitation to the National Health Service Health Check programme.

The Newcastle site focused on the recruitment of participants at risk of dementia from low-income and ethnic minority groups. This was to understand if limited knowledge of dementia persisted among these groups.^13 14^ The Nottingham site focused on recruiting people from South Asian, African and Caribbean communities.

### Data collection

MH, AA-O and MBr conducted focus groups (FGs) remotely via Zoom or MS Teams, or in person (eg, in community-based settings) depending on group preference, guided by data saturation. Sessions were audio recorded. Where feasible, specific groups were run in languages other than English or were single-gender sessions in line with communities’ preferences. Each FG did not exceed 2 hours. A £20 voucher was offered as a token of appreciation to each participant for attendance.

We collected data using a combination of FG and task group methodology as previous research suggests awareness and understanding of dementia is poor among the groups we were engaged with.^13 14 18^ We explored dementia as a concept using FG principles (open questions without any presentation of material to assess baseline knowledge), before employing task group methodology where discussions were based on material presented to the groups. We used a semistructured approach which enables preplanned topics to be covered, while affording ample opportunity for the facilitator to explore additional points brought up in groups in more depth (see online supplemental material 2 for more details on topics of discussion).^22^ This methodology is particularly useful when there are likely to be differences between the participants and grouping people based on criteria such as ethnicity may help individuals feel more comfortable about participating, resulting in the collection of more valid data.^22^

### Patient and public involvement

We engaged with existing patient and public involvement representatives (from the Dementia Care Community Group in Newcastle) and local community gatekeepers at both sites to help design, refine and pilot approaches to recruitment, FG delivery and the content to be delivered using task group methodology. We also sought input from these stakeholders on topic guide development to ensure that the language was appropriate, and questions were clear. Public engagement enabled the research team to understand different cultural norms and practices across communities and how to consider this to encourage participation and ensure approaches were culturally sensitive and inclusive. Additionally, two of the group facilitators (AA-O and MBr) attended Cultural Competence Training run by The Centre for Ethnic Health Research at Leicester University (https://ethnichealthresearch.org.uk) which enhanced cultural competency among the research team.

### Data analysis

Audio recordings were transcribed verbatim, checked for accuracy and personal identifiers were removed. We analysed data using the framework approach, which is a hierarchical, matrix-based method developed for applied research.^23^ This approach enabled us to map thematic differences/similarities within and between groups, for example, by site, SES or ethnicity. Researchers (MH, AA-O, MBr, RB and MBa) coded according to a priori themes (based on aims and task group discussion topics) and inductively. Initial transcript readings facilitated familiarisation and initial code generation. Further reading and immersion resulted in more substantive themes and subthemes, resulting in the generation of an analytical framework. Data were indexed according to the identified thematic framework. A subsample of data was double-coded to ensure the validity of interpretations. Themes were discussed and agreed on between the research team, allowing clarification of the final framework that was then applied across all the transcripts. Data were charted according to each theme to facilitate interpretation, synthesis and reporting.

###  Ethical approval and informed consent

All participants were provided with a Participant Information Sheet via email or in person (via gatekeeper or attending local community settings), ensuring that the participant had sufficient time to consider participation. Gatekeepers helped interpret for members who had English language difficulties to aid understanding. All participants provided written informed consent, completing either a digital copy or paper copy of the consent form, before participating in a FG.

## Results

### Participant demographics

12 FGs, comprising between 6 and 17 participants per group, were conducted with 147 participants across the two sites: 8 FGs from Nottingham (n=105) and 4 FGs from Newcastle (n=42). 62% of participants were female (n=91). Participants were from diverse ethnicities with 37% black or black British, 24% Asian or Asian British, 20% white, 9% not provided, 7% Arab and 1% other ethnicities. Education levels varied, with 21% having General Certificate of Secondary Education, and 16% holding a bachelor’s degree. See table 1 for participant characteristics.

**Table 1:** Characteristics of participants

Category	Description	Participants n (%)
Sex	Female	91 (62)
	Male	46 (31)
	Not provided	10 (7)
Ethnicity	Black or black British (Caribbean, African or Other)	55 (37)
	Asian or Asian British (Indian, Pakistani, Other)	36 (24)
	White (British, Irish, Other)	30 (20)
	Not provided	13 (9)
	Arab	11 (7)
	Other	2 (1)
Education	GCSE level	30 (20)
	A-level	19 (13)
	Bachelor’s degree	24 (16)
	Master’s degree or above	9 (6)
	No formal education	16 (11)
	Not provided	49 (33)

No. of participants (N)=147.

Mean age = ∼63 years.

GCSE, General Certificate of Secondary Education.

### Thematic framework

Four overarching themes were generated, with corresponding subthemes as shown in table 2. The results are presented under these themes and subthemes (for supporting quotes see online supplemental material 3). The preliminary analysis looked at within and between-group differences. Findings were consistent across the sites; however, some ethnicity-related differences were noted and have been reported within the corresponding themes.

**Table 2:** Thematic framework

Overarching themes	Subthemes
Knowledge and awareness of dementia	Knowledge about dementia, the symptoms and risk factors
	Approaches to raising awareness
Attitudes towards risk prediction	Limited knowledge
	Psychological distress
	Empowered to make changes
Barriers to accessing dementia services	Challenges accessing healthcare services
	Fear and stigma
	Denial
	Misconceptions about ageing and memory loss
	Language barriers
	Cultural and religious beliefs around dementia
	Cultural approaches to caring for elders
Service improvement recommendations	Workforce training needs
	Improve language accessibility
	Engagement through community-based infrastructure

#### Knowledge and awareness of dementia

##### Knowledge about dementia, the symptoms and risk factors

Participants had a general understanding of dementia, symptoms and risk factors; however, knowledge of dementia services and how to access support varied across FGs. Participants from ethnic minority groups felt that dementia was not well understood among their communities. Some attributed this to no direct translation of the term in other languages and the stigma associated with dementia.

All participants recognised memory problems as being a key feature of dementia. Other symptoms mentioned by participants included confusion, headaches, change in temperament, communication difficulties and mood changes.

Participants discussed several factors that may increase one’s risk for dementia and recognised that some were modifiable. These risk factors included lifestyle, substance misuse, physical trauma (stroke, brain injury from physical sports), psychological trauma, environment, pre-existing health conditions, family history of dementia, education, age, lack of sleep and hearing loss. Additionally, a lack of faith or spirituality was seen to be a cause of dementia by participants who belonged to a faith or religion (see online supplemental material 4 for a more detailed Framework Analysis of risk factors).

##### Approaches to raising awareness

Participants highlighted a need for more information on dementia symptoms, how dementia is assessed, what risk prediction is and what treatment is available to be accessible for all communities. Participants advocated various ways to share this information, including education campaigns, using videos or posters and ensuring information is available in different languages. Some participants also suggested reaching out to well-known individuals, such as community or faith leaders who could reach more people to share information. Participants also suggested that improving knowledge and awareness among their communities may help to overcome the stigma associated with dementia.

### Attitudes towards risk prediction

#### Limited knowledge

Knowledge of risk prediction in dementia was low across the FGs. Participants discussed how they were unaware of the term risk prediction, how it was used and that tools are being developed to predict dementia risk. Not having this knowledge was perceived as a barrier to understanding the benefits of predicting risk. Efforts to inform people of low SES and ethnic minority groups about dementia risk prediction tools are warranted.

#### Psychological distress

Some participants were unsure about whether they wanted to know their risk for dementia. Participants also discussed whether there was anything that could be done to reduce risk or prevent dementia from happening. If there was nothing that could be done, participants shared that it may not be worth knowing one’s risk as it would likely cause psychological distress.

#### Empowered to make changes

Participants who were interested in understanding their risk for dementia better discussed how it would empower them to change their lifestyle to reduce their risk, though it was highlighted that healthcare professionals would need to provide appropriate advice and support for this to happen. Participants also felt knowing their dementia risk would prompt them to plan for their future and make decisions ahead of time about their health and care, should they go on to develop memory problems.

### Barriers to accessing dementia services

#### Challenges accessing healthcare services

Participants reflected on their own experiences accessing primary care services for various health challenges including dementia and dementia-specific services. For primary care services, challenges included not being able to book an appointment with a general practitioner (GP), a lack of continuity with GPs, including not seeing the same one for subsequent appointments and limited consultation times not giving participants sufficient time to raise the issues they wanted to address with their GP. Participants with experience of accessing dementia services found the lack of regular follow-ups with memory clinics postdiagnosis was very challenging and left them unsure of what happened next.

#### Fear and stigma

Fear was seen to hold people back from undergoing memory assessments or accessing support. Participants discussed the ways fear presented itself, including dementia being scary, fear of the hospital and the fear of how one’s family will cope if they were to have dementia. Some participants also discussed a fear of how being misdiagnosed, not specific to dementia, may hold people back from seeking support. Concerns of misdiagnosis were primarily raised by participants from ethnic minority backgrounds.

All participants felt that there was still a stigma attached to a dementia diagnosis, particularly within ethnic minority communities, and that this may hinder some from accessing the appropriate health services. Dementia is still seen as a mental health problem in some cultures. Also, talking about dementia is a taboo subject, and people may not want to admit there is a problem. Participants discussed how the stigma associated with dementia may result in individuals being neglected by members of their own family, their relatives and their community, contributing to a reluctance to access services.

#### Denial

Participants discussed how individuals and even their families may be in denial about their memory problems. Reasons for denial included feeling like the individual is losing their independence and the thought that someone may take over their life and control them. Participants from African communities highlighted how black men are proud and may struggle to admit they may have problems with their memory, so they may choose to suffer in silence instead of seeking support. Denial of memory problems was not specific to ethnic minority groups, with participants from low-income groups also reporting this issue.

#### Misconceptions around ageing and memory loss

Participants, from South Asian and black ethnic minority groups, talked about how memory problems were viewed by their communities as being a normal part of ageing. They highlighted how people within these communities viewed memory loss as common in later life and ‘it happens to everyone’. Participants suggested these misconceptions around ageing and memory loss may contribute to delays in diagnosis as people may put off seeking help until the memory problems are much more severe.

#### Language barriers

Language barriers were reported to also contribute to delays in diagnosis and problems accessing appropriate health services. Participants highlighted that language barriers mean individuals, including those with dementia, are unable to express themselves. There is a need for translators to make it easier for people to communicate their problems. One participant reflected on their experiences with healthcare professionals and noted that they were more likely to be examined properly with a translator or family member present at the appointment to translate for them, compared with when they attended appointments alone.

#### Cultural and religious beliefs around dementia

In addition to various barriers to dementia services, FGs where participants were from ethnic minority groups and/or identified as belonging to a faith discussed specific cultural and religious barriers, which may hinder access to dementia diagnostic support and health services.

Cultural beliefs or cultural stigma may affect how someone with a diagnosis of dementia is perceived by those around them. One participant discussed how once there is a formal diagnosis of dementia, the individual’s peers, their community and relatives are likely to avoid them. Religious beliefs around the causes of dementia may also contribute to low uptake of dementia services and accessing diagnostic support. One participant’s view was that dementia was a result of not engaging in spiritual practice. Another participant suggested that dementia may be caused by not praying enough. Participants also discussed how in some faiths, prayer and religious practices are seen as medicine, so individuals may consult their faith first for remedies for their health. Muslim participants discussed how in Islam illness is also viewed as a grace from God and their faith teaches them to accept this.

#### Cultural approaches to caring for elders

Diverse cultures have differing practices when it comes to the care of elderly family members. In Black, South Asian and Arab cultures, families often look after their elders and create a network or support system around them to protect them. This may include shielding their symptoms from relatives, peers and the wider community to protect them from the stigma associated with dementia within their culture. This may also prevent the individual from accessing dementia diagnostic and support services as the family may decide against it and to look after the individual.

### Service improvement recommendations

#### Workforce training needs

Participants suggested three key training needs for healthcare professionals to improve the dementia diagnosis process and access to reliable information about support and care available. (1) Cultural competency training, (2) Dementia awareness training, (3) Communicating dementia information to patients from diverse backgrounds.

Participants stressed that cultural competency training was highly important to ensure that people from ethnic minority groups are treated fairly, have access to quality services and can access dementia services at the appropriate time.

A need for further training to ensure that healthcare professionals can adequately support patients who exhibit signs of dementia was noted by participants, as a common shared experience was that healthcare professionals lacked understanding of dementia.

Participants discussed the need for accessible information on dementia in different languages and formats for diverse groups. Participants also reflected on how communication challenges with healthcare professionals, particularly within a primary care setting, meant they hesitated to seek further medical advice. Some participants suggested that training for healthcare professionals on how to effectively communicate dementia information with patients from ethnically and linguistically diverse groups is needed.

#### Improve language accessibility

There is a need to improve language accessibility across dementia services and the diagnosis process. This should involve translating written materials such as patient information leaflets or having better access to translators during medical appointments. Additionally, participants have recommended having translators during FGs and engagement events where medical information is shared would increase engagement.

#### Engagement through community-based infrastructure

Community engagement was seen as key to raising awareness of dementia and improving earlier diagnosis. Participants suggested various approaches to engage with communities, such as setting up clinics within community hubs where people can get more information and the use of communication channels such as WhatsApp to share information and details about community engagement events. Participants also felt the use of the task group format used in this study to share information would be an effective way to engage with different community groups to share information about dementia. One participant suggested healthcare professionals and researchers should join in events held for different festivals across communities to share information about health and related services. Participants also suggested connecting with community leaders as they can reach more people within the community.

## Discussion

All participants indicated awareness of dementia and associated symptoms while highlighting concerns about healthcare professionals’ knowledge of dementia and stressing the need for better access to reliable information about dementia. Participants’ understanding of dementia risk factors was comprehensive. Participants belonging to a faith or religion viewed the lack of faith or spirituality as a potential risk factor for dementia, which is a noteworthy finding. Attitudes towards dementia risk prediction were cautiously optimistic, and the use of such tools was viewed as empowering if accompanied with advice to make changes to reduce risk. Barriers to implementing dementia risk prediction tools were a lack of awareness among diverse groups and healthcare professionals and the potential psychological impact of a high-risk result. Barriers to accessing dementia services included challenges accessing healthcare services, stigma, denial, language and cultural and religious views about dementia. Recommendations for service improvement included workforce training, language accessibility and community engagement.

Challenges in accessing reliable information persist across diverse communities and impact the ability to navigate dementia services and access appropriate support.^24 25^ Concerns around limited knowledge of dementia among healthcare professionals have been reported previously.^25 26^ Participants from religious backgrounds viewed engaging with religious or spiritual practices and not praying enough as risk factors for dementia. Participants shared the idea that engaging in prayer and religious practices was protective against dementia, and the reason one may develop dementia is because they did not engage enough with religious practices. Previous work exploring knowledge of dementia among South Asian communities noted similar findings that dementia was seen to be caused by religious or cultural factors such as a lack of faith, punishment by God or evil spirits.^18 26 27^ Healthcare practitioners need to acknowledge and understand the role religious and cultural practices and beliefs can play in approaches to accessing dementia services among patients from diverse backgrounds.

Previous smaller-scale qualitative studies have demonstrated varied knowledge of dementia risk factors and risk prediction,^14 28^ demonstrating that limited knowledge of risk prediction is an issue across different communities. Our work extends these findings, showing knowledge of risk prediction is also low among low-income and ethnic minority groups. Participants suggested various approaches to improving knowledge and awareness of dementia, risk prediction and dementia services such as ensuring information is available in different languages and connecting with community leaders to share information within their communities. The latter is a commonly cited approach by ethnic minority groups as a preferred way of disseminating health information among diverse communities across various therapeutic areas such as dementia, COVID-19, mental health, maternal health and precision medicine.^29^,^34^ Participants also emphasised how tailored community engagement was key to improving awareness of dementia and associated risk prediction tools while helping to reduce the stigma across communities.

### Recommendations

We present three key areas for action to improve awareness of dementia risk prediction and service access for diverse groups.

1. Cultural sensitivity training for healthcare professionals to improve awareness of how dementia and risk prediction are perceived by different cultures and faiths.
2. Better access to translators and information in different languages and formats for linguistically diverse groups and those with differing literacy abilities.
3. Engaging with communities to co-create meaningful and effective strategies to improve knowledge and awareness around dementia risk prediction and facilitate increased access to services and support.

### Strengths and limitations

Historically, involvement of ethnic minority groups in dementia research has been low.^35 36^ Evidence shows that engagement between research and diverse communities is challenging, and the voices of these communities are often under-represented.^37 38^ In this study, we engaged with diverse communities and recruited a large sample of participants representing low SES and ethnic minority groups. However, we do acknowledge that some of these groups are better represented in our sample than others. Despite the small numbers for some groups, the key strength of this work is that we were able to engage with these groups and they participated in the study.

We considered the needs and cultural norms of the communities we engaged with by running single-gender FG sessions where this was preferred and providing language support via translation of materials and interpretation during FGs. The use of task group methodology contributed to meaningful community engagement practices through reciprocity whereby the groups received information while participating in research, enhancing researcher-community relationships.

We found no site-related differences in our findings, which enhances the transferability of the findings to similar diverse groups in different regions of the UK. However, we did not recruit participants from other ethnic minority groups which experience notable inequalities such as Gypsy, Roma, Traveller and Bangladeshi communities. Future research needs to explore the barriers for other ethnic minority groups and identify service improvement recommendations to further improve accessibility and inclusivity of dementia services across the UK.

## Conclusion

This study provides novel insights into current levels of dementia knowledge, attitudes towards risk prediction and perceptions of the impacts of risk prediction tools among socioeconomically and ethnically diverse populations in England. Barriers to health services for low-income and ethnically diverse groups, alongside service improvement recommendations, offer a starting point for service providers to develop culturally aware and inclusive dementia services at prediagnostic and postdiagnostic levels.

## Supplementary material

10.1136/bmjopen-2024-092370 online supplemental file 1

10.1136/bmjopen-2024-092370 online supplemental file 2

10.1136/bmjopen-2024-092370 online supplemental file 3

10.1136/bmjopen-2024-092370 online supplemental file 4

## Data Availability

Data are available in a public, open access repository.
