# Supplementary material for: Qualitative study exploring knowledge and attitudes towards dementia risk prediction, barriers to dementia services and service improvement recommendations with diverse populations in England
Source: BMJ Open. 2025 May 30;15(5):e092370. doi: 10.1136/bmjopen-2024-092370 (PMC12128399; doi:10.1136/bmjopen-2024-092370)
Supplement: online supplemental file 2 [file bmjopen-15-5-s002.pdf]

University of Nottingham
UK | CHINA | MALAYSIA

Topic Guide

OBJECTIVES

- To understand knowledge around dementia and testing for dementia;
- To explore knowledge about what participants think are the key risk factors for dementia and which could be used to inform how we could test/screen for dementia
- We will use findings from our discussions to inform the development of tools that could be used to help predict who may be more likely to be diagnosed with dementia so that we can either prevent or treat earlier .

INTRODUCTION

- Check individual has read the information sheet.
- Explain the purpose of the discussion and how the results will be used.
- Explain how long the focus group discussion will take.
- Ask participant if they have any questions.
- Check consent form has been signed and check still happy to take part.
- Statement on confidentiality, right to withdraw consent, recording of the interview.

We would like to reassure you that all data relating to yourself will be kept strictly confidential by the research team. The recording of this interview and any quotes used in study reports will not identify you in any way. You will be assigned a unique study code that will not identify you. Your participation is entirely voluntary and you are free to withdraw at any time without giving a reason.

BACKGROUND INFORMATION

(BEFORE PRESENTATION 1): Understanding and knowledge about dementia

- What comes to mind when you hear the word ‘dementia’?
- What do you think are the risk factors for dementia? And how these can relate to our health?
- Can you tell me about your personal experience with dementia? (e.g., within family or social group)
- To what extent has that affected your feelings about dementia and its risk factors?
- What is your understanding of risk prediction in relation to health conditions?
- What is your understanding of risk prediction tools in relation to health?

<u>Presentation 1</u>: Dementia, how we diagnose it & the importance of including ethnic minority groups

Group discussion 1

Attitudes towards dementia and risk factors

- What comes to your mind after watching/listening to the presentation?
- What are your views about dementia?
- What are your views about dementia risk factors?

<u>Presentation 2</u>: Dementia prevention & risk assessment

Group discussion 2

- What comes to your mind after watching/listening to the presentation?
- What are your views about dementia risk assessment?
- What are your views about dementia treatments?

Barriers to dementia risk assessment

- Can you think of any challenges for assessing the dementia risk?
- What can the healthcare system do to reach and engage communities including their own? **(Explore within the context of their particular ethnic group)**
- How education on this should be done?

Facilitators of how dementia risk assessment may work

- If a tool were to be developed, how would we ensure people are aware of it?
- What are your communities needs for such a tool?

Views on dementia risk assessment tools

- What are your views about dementia risk assessment tools?
- How to make it feasible in your community?
- What are the challenges for you?

Final discussion

- What have you learnt about dementia from this session?
- What have you learnt about dementia risk factors from this session?
- Is there anything that can be done to improve:
  1)knowledge of dementia risk factors
  2) dementia risk assessment tools

University of Nottingham
UK | CHINA | MALAYSIA

Closing Remarks

- Is there anything you would like to add that we have not already covered?
- Reassure about confidentiality
- Thank participant for their time – offer voucher incentive.
