# Supplementary material for: Qualitative study exploring knowledge and attitudes towards dementia risk prediction, barriers to dementia services and service improvement recommendations with diverse populations in England
Source: BMJ Open. 2025 May 30;15(5):e092370. doi: 10.1136/bmjopen-2024-092370 (PMC12128399; doi:10.1136/bmjopen-2024-092370)
Supplement: online supplemental file 3 [file bmjopen-15-5-s003.pdf]

Theme	Sub theme	Quote
Knowledge and awareness of dementia	Knowledge about dementia, the symptoms and risk factors	“I think as I said that loss of memory, not full memory, but the present memory. They remember the past very well. But the present, dementia people, they don't remember. That's my understanding” – Muslim women focus group<br><br>“And I think we have to change our diet as well. It's part of it. Because we eat too much rice and too much oil, too much fatty food. Because there is a feast, our Muslim feast last night, I was watching the way the Senegalese were cooking. Mama Mia. The number of things they put in that food. Definitely if you eat it, you will never feel healthy” African English focus group<br><br>“Another thing is that if you live alone, have less interactions with people can also be a risk factor. Being socially active with everyone is important” – FG4 (Asian, Indian and Pakistani).<br><br>“Okay. Well I’ve got three. A lack of exercise, you know on a whole lifetime issue, which I think is hugely important. Smoking. Drinking” – FG2 (White, Pakistani, Mixed White and African).<br><br>“Male: Well, you’ve already touched on, but you didn’t actually mention head injuries.<br>Facilitator: Yes. Stroke.<br>Male: And also-<br>Facilitator: Stroke, sorry, yes. A good point, stroke.<br>Male: … activities. And somebody mentioned football and this research has now been done into footballers, where heading footballs from years ago when they were much heavier.<br>Female: And boxers.<br>Male: And also boxing.<br>Female: And rugby.<br>Female: Yes”<br>FG1 (White, British/Irish/Other)<br><br>“Participant 1: Mental health.<br>Researcher 1: Yes, in what way?<br>Participant 1: If you are isolated and you feel that you are doing okay, but then you may end up with an illness and become more isolated I think the risk factor with dementia would go up” FG1 (White, British/Irish/Other).<br><br>“There is this one I didn’t like because I didn’t go to university. They say if you are better educated, the better

Knowledge and awareness of dementia		you are educated it can reduce your risk. I think there probably is some truth in that" – Newcastle FG2 (White, Pakistani, Mixed White and African).<br><br>"I think when you got lots of problems like arthritis or chronic pains, and fibrosis, diabetes, pressure. The old pressure comes, all these pressure. And that makes you more forgetting or, don't remember the things. And don't know what they're doing" – FG7 (Asian Muslim women's group).<br><br>"Usually, the one that I've seen is people who are in their 80s approaching their 90s, that's when it develops most of the time" – FG8 (Asian, Pakistani men's group).
	Approaches to raising awareness	"We need education campaigns. We needed volunteers to translate into other languages even if the tools were there. We, as ethnic minorities, have a language barrier. Translators may cost NHS a lot of money, so we need volunteers. This will help a lot of patients. This will educate patients and also helps them. We have to encourage others to volunteer and help the communities long-term" – FG6 (Arab men and women's group)<br><br>"They can reach out to well-known people in each community, and they will tell others. You will not be able to reach everyone, but eventually, the information will reach most people. You have to meet up with people. But people are afraid of participating themselves. They have to know someone that they trust." – FG5 (Arab women's group)<br><br>"I personally think that there should be hubs set up around the city, especially for diagnosis of dementia, where you can get all the information which you won't get from the doctor because he doesn't have the time to do it. If you've got a hub where you've got professional people who can do the risk assessment, who can tell you what to expect. Tell you what sort of dementia you've got or your family or loved ones, friends. Yes. And for the resources to be available there for you to access. Surely that would be a great help to everybody." – FG8 (Asian, Pakistani men's group).
Attitudes towards risk prediction	Limited knowledge	"There's not have enough knowledge for the subject." – FG8 (Asian, Pakistani men's group).<br><br>"Can you explain the word? What do you mean by risk prediction?" – FG11 (African men's group).
	Psychological	"Personally, I think I would want to know my risk factors for similar reasons, really, that I would want to plan

Attitudes towards risk prediction	distress	ahead and try and embrace it. But I know from my own family, that's not always possible, some people can and some people can't." – FG2 (White, Pakistani, Mixed White and African).<br><br>"Participant 3: What I was going to say was, my friend who is 69, has just been diagnosed with Parkinson's, and he's seriously depressed now with that, and he was never a depressed person, and he's anxious about the whole thing. And I feel as if I was destined to get something, and if I thought I was, it might just put me into a spiral. It might, it might not-<br>Facilitator: That's understandable.<br>Participant 3: ...but some people it might, it might not, it depends on your mindset, doesn't it. If you think you can do something about it, then it's worth a bash, but if you thought you were 60% likely, well what's the point, you might as well give up. I wouldn't, but..." – FG1 (White, British/Irish/Other)
	Empowered to make changes	"Participant 9: I feel like I would want to plan around it if I knew, because I know – well, I don't know, but at the minute there's very little that they can do for it – so I would like to plan my future, so if I know that, then I would want to plan." – FG1 (White, British/Irish/Other)<br><br>"I think when you say risk predictions, I think you have to look ahead. And you have to say you've got the person then, all right, they might be all right, but you have to look to the future. And you have to put actions in place to say, all right, if so-and-so's there and at the moment she's fine, maybe in a week or two months' time, she might be unsteady on her feet or it might lead to a heart attack. So you have to put actions in place to maybe not stop it but to prevent it being worse than it could be." – FG9 (African Caribbean group 1)

Barriers to dementia services	Challenges accessing health services	“Now these day it is very hard to get an appointment.” – FG4 (Asian, Indian and Pakistani).<br><br>“There used to be thing called a family doctor, you will see same doctor no matter what. But in a way they would know your history because they're seeing you continuously. But now every time you're in, different doctor.” – FG7 (Asian Muslim women’s group).<br><br>“My GP’s got a notice up saying, “Ten minutes, one patient, one problem,” and I forget what the other bit is. And that’s what they’ve got actually sticking on the wall. So, have you got time to discuss all that in ten minutes? No.” – FG1 (White, British/Irish/Other)<br><br>“Participant 4: That’s what I’m talking about. It’s the follow-up.<br>Participant 3: Yes. I didn’t have anything when husband was diagnosed. Nothing.<br>Participant 6: Did they not talk to you at the Memory Clinic?<br>Participant 3: Yes, and then they said, “His diagnosis is Alzheimer’s.” I was discharged.<br>Participant 6: But no, “And this is what he now needs to continue to do.”” – FG2 (White, Pakistani, Mixed White and African).
Barriers to dementia services	Fear and stigma	“Yes, I think a bit, yes, a bit of stigma. Same as mentally, isn't it? It's a similar thing, isn't it? People don't want to accept that they've got problems with mental problems. I think the same thing.” – FG7 (Asian Muslim women’s group).<br><br>“So often the aspect of family becomes really important. If you get a certain age and you're not surrounded by family, that is when stigmatization, and names, and name calling, you be called a witch or wizard because there is the memory loss that you're doing things in what is considered abnormal. Some of them are going out at night because they don't really know that awareness and then they get called names and then they're called wizards or witches.” FG11 (African men’s group)<br><br>“But the fear of going into hospitals, we are misdiagnosed. If they see like somebody of colour or something who's behaving erratic or somebody, they don't put it as manic depressive or something. First label they're giving them, they're suffering from schizophrenia. It’s getting better now. But before, people were very fearful of that being misdiagnosed and ended up in a mental institute for being misdiagnosed.” – FG9 (African Caribbean group 1)

Barriers to dementia services	Denial	“RESPONDENT 4: But the dementia people who got the dementia, they don't accept that they got any problem.<br>RESPONDENT 2: Yes. Yes. They are always arguing that no; I got nothing wrong with me. It's you. You are making it. Nobody like it. Nobody like it. They want to argue about it.<br>RESPONDENT 3: Like she said, the early, those people who with the early dementia, they are the ones who don't want to accept. So your husband doesn't want to accept that he's got dementia.<br>RESPONDENT 4: He still he doesn't want to accept, although he is taking medicine, but he says no, there is nothing wrong with him.” – FG7 (Asian Muslim women’s group).<br>“Yes. And for them to say they've got it, they're going to think someone's got to take over in their life and controlling them, and I think that's the main problem.” – FG9 (African Caribbean group 1)<br><br>“ I was saying about black men, they don't want to admit some things and they would rather stand like a man. So maybe they're going down, they will not admit…” – FG9 (African Caribbean group 1)
	Misconceptions about ageing and memory loss	“Maybe they don't consider when they start forgetting things. And like the symptoms you said, maybe they don't consider it. That this is part of getting old.” – FG7 (Asian Muslim women’s group).<br><br>“What happens if somebody keeps so forgetting and they take it, it happens. You are getting old. It's a normal thing. That is a very big factor. So, I would say in one word it is a lack of awareness and acceptance by us, yes, we are going through dementia if some individual person in the family or anything.” - FG8 (Asian, Pakistani men’s group).

Barriers to dementia services	Language barrier	"Participant 7: I think sometimes language barrier is, sometime other is not able to express themselves, properly explain themselves.<br>Interviewer: Yeah.<br>Participant 5: If you can't explain yourself, then it is difficult for you.<br>Participant 3: Difficult.<br>Participant 8: If there is no language barrier for you then you can explain.<br>Interviewer 6: A translator is required.<br>Participant 2: Many people need a translator to explain their problem.<br>Interviewer 4: Right." – FG4 (Asian, Indian and Pakistani).<br><br>"I think language will be. It's not in my case, but in some cases, there will be a big barrier. Language is a big barrier to explain. When you got dementia, especially, you can't…" – FG7 (Asian Muslim women's group).
	Cultural and religious beliefs around dementia	"But in terms of culturally for a time you get that formal diagnosis, in terms of dementia, you are somewhat avoided by lots of people, even within your own community, and within your family." – FG9 (African Caribbean group 1)<br><br>"I think they might be abandoned or something like that, or other families maybe neglected in community. Stigma." – FG10 (African Caribbean group 2)<br><br>"Yes, I think a bit, yes, a bit of stigma. Same as mentally, isn't it? It's a similar thing, isn't it? People don't want to accept that they've got problems with mental problems. I think the same thing. I think people start ignoring you as well. People think of he or she is, they like that and not paying much attention to what she was suffering and what she's saying." – FG7 (Asian Muslim women's group).<br><br>"It helps a lot, looks like if you're a good Muslim, you don't get depressed at all because of your belief, you never get depressed. Because the Allah says in the Quran, when you remember Him a lot, you're free for every disorder, any stress. So as a Muslim, I don't say that no Muslim won't be depressed, but it's very rarely Muslim have that particular. When you go back to Allah, all the stress will…If you have depression, you go back to Allah and you relieve. Like if you have a sickness, you remember the medicine, it cures your sickness. So, if you have depression, you went back to Allah to remember Allah, depression will go.<br><br>What he's saying is that like the faith that you have in Allah strengthens you mentally and emotionally that

Barriers to dementia services		whatever happens, you feel, might be something decreed on me, so I'll take it in good faith instead of soaking myself into this problem and creating more problems for myself. I'll accept it. healthy eating and all of those, change your lifestyle, which Islam in fact encourages you to do. Then when you finish your evening prayer, you have to have a walk around before you go to bed. It teaches you all of those things. This is the lifestyle of our prophet. And if you follow that way, it helps us a lot. But this is very difficult to explain to the Eurocentric or Western educated person because you would say it's not scientifically proven, it has to be scientifically tested. But it's a different thing. And these are very important factors that come into play when you come to this kind of disease." FG11 (African men's group)
	Cultural approaches to caring for elders	"I think culture is a big thing with dementia. Like the Asian community, they're very close and they do things in certain ways, so they don't open up to what is outside, because they have maybe member of their family, the elders might be able to talk to, because quite often the speak in groups." FG10 (African Caribbean group 2)
Service improvement recommendations	Workforce training needs	"I think it's both. I think it's both. I think they don't know enough about the different, different cultures because that's not part of their training. That isn't part of their training. And then they don't know enough about the specific illnesses. They know a bit, but they don't know enough." – FG9 (African Caribbean group 1)<br><br>"I think we probably need to have more culturally competent staff members that we need to outreach of this nature to actually come to centers like here and actually engage the community like this and then we could have a conversation like we are having with you. And, of course, that will take the healthcare professionals to actually come out of these slightly comfort zones and actually reach out to communities because there's that lack of knowledge within specific communities about the disease or the condition. Then we need to reach out to those communities." FG11 (African men's group)<br><br>" My point is that going to the GP or coming to the point where you are suffering from dementia, people need to be given more services, more communication, more time or resources, before they get to that point." – FG8 (Asian, Pakistani men's group).
	Improve language accessibility	"Yes. I think I just add on. Using the social media with different languages. You know, in all languages, it'll be easier for people to access because social media, they use that one." - FG8 (Asian, Pakistani men's group).

Service improvement recommendations		"Someone who can translate it in our language whereby maybe they will have a lot of people who would be thinking of, oh, if I go there, even if I can't understand English, I'll be able to understand the other language. So that would maybe convince them more to come." – FG12 (African women's group)
	Engagement through community-based infrastructure	"The healthcare needs to identify what groups are in the community that have influence. And then when they communicate that we'd like to have a focus group or we'd like to do an awareness session, then those strategic groups can be informed and then they'll inform whoever's linked to them." – FG9 (African Caribbean group 1)<br><br>"Yes. In the community that would say, right, there's a member clinic once a month at some, you know, wherever. If you don't have anything, you want help. Attend the clinic, somebody will see you and advise you where to go next." FG10 (African Caribbean group 2)<br><br>"And we have a WhatsApp group that if he puts it in the WhatsApp and talks about what is going on, I think, is that him? So, if he gets involved, I think and has the message in the WhatsApp group, I think a lot of people would attend because he's well known. Yes." – FG12 (African women's group)<br><br>"We know national health is overburdened with everything. But this service could be provided better by the community, by their ethnic minority within, you take example as our group here. We talk all our languages, which another, easy to understand. And I'm sure everybody will agree with me. I, myself personally, if I don't attend this session for three or four days, stay home, I feel I'm missing something. It starts affecting my health." – FG8 (Asian, Pakistani men's group).<br><br>"No, B (name of community leader) he's in our community. DC (name of person) Mr F (name of person) ... You will see him, he wears glasses. Yes. He's very, very active in our programs. And if he sorts of reached people. No, he's like a community leader. He organizes things and lets people know what is going on so we can get a bit of a congregation for people to come and one like G (name of participant) and I, as I said, D (name of a person) and T (name of a person), can enlighten." - FG12 (African women's group)
