# Supplementary material for: Qualitative study exploring knowledge and attitudes towards dementia risk prediction, barriers to dementia services and service improvement recommendations with diverse populations in England
Source: BMJ Open. 2025 May 30;15(5):e092370. doi: 10.1136/bmjopen-2024-092370 (PMC12128399; doi:10.1136/bmjopen-2024-092370)
Supplement: online supplemental file 4 [file bmjopen-15-5-s004.pdf]

Supplementary material 2

Framework analysis to identify additional risk factors to dementia in consultation with ethnic minority and low-income groups.

Methods

Data collection

Task Group methodology is particularly useful when there are likely to be differences between the participants and grouping people based on criteria such as ethnicity may help individuals feel more comfortable about participating, resulting in the collection of more valid data (10).

Examples of key areas for discussion were: (1) limitations in knowledge of dementia and its risk factors; (2) key risk factors for dementia as highlighted by our reviews of risk prediction models and suggested by group members; (3) individual/group differences in perceived risks; (4) barriers and opportunities for accessing healthcare; (5) issues around staff training and how and where risk should be presented; (6) ethical implications in terms of risk assessment and management; and (7) perceived benefits/harms.

Patient and public involvement

Our study was guided by engagement with collaborators and partners from both Nottingham and Newcastle with whom we have previously worked to engage underserved communities, on various projects. They helped design and pilot the approach (focus groups and task group methodology) in addition to the topic. They also informed the development of the grant proposal, and guided the data collection, analysis, and dissemination.

Reflexivity

The research study team was comprised of AAO, MH, RB, MB, NQ, LR, RA, BS who were from diverse genders, ethnicities, educational and professional backgrounds with varying migration statuses across the team. Colleagues' professional backgrounds included pharmacy, public health, mixed methods research, psychology, general practice with previous research experience and training. As a team from diverse personal and professional backgrounds, we reflected on how our own experiences may have affected both data generation and interpretation, through a process of active reflexivity. Field notes were also made after the focus/task groups to complement team discussions. This was particularly important to consider for some of the focus groups - two of these were conducted in Arabic, as one of the researchers were native speakers of these languages. This may have helped positively engage

with participants and create a more natural setting for data gathering, but we also acknowledged the importance of trustworthiness and consistency in this process. This work was also guided by inputs from the PPI, which includes members from communities of various ethnic, migrant and socioeconomic backgrounds. In other cases, for example for those researchers medically trained, it was important to maintain a focus on data collection rather than a more clinically-nuanced encounter to ensure consistency in data collection.

Results (dementia risk factors framework analysis)

To explore participants' knowledge of dementia-related risk factors and identify potential new risk factors to inform the development of a new risk prediction algorithm, framework analysis was used to analyse data about dementia risk factors.

Across all focus groups participants discussed risk factors for dementia. Through these discussions, we identified several themes and sub themes relating to lifestyle, trauma, environment, existing health conditions, family history, faith/spirituality, level education, lack of sleep, age and hearing loss (See table 1).

Supplementary Table 011 Framework for Dementia Risk Factors

Theme	Sub themes
**Lifestyle**	• Unhealthy diet<br>• Lack of exercise<br>• Limited hobbies / interests<br>• Lack of social activities /social isolation<br>• Substance use (smoking/drugs/alcohol)
**Physical Trauma**	• Brain injury<br>• Stroke<br>• Caused by contact sports
**Psychological Trauma**	• Traumatic life events<br>• Stress<br>• Anxiety

	• Depression<br>• Worry / overthinking
**Environment**	• Pollution<br>• Socioeconomic disadvantage<br>• Varied access to quality healthcare services
**Existing conditions**	• Existing medical conditions e.g. hypertension<br>• Existing mental health conditions e.g. depression
**Family history**	• Family history of memory problems<br>• Genetics
**Faith / spirituality**	• Lack of faith<br>• Not praying or engaging in spiritual practice
**Level of education**	
**Lack of sleep**	
**Age**	
**Hearing loss**	

Lifestyle

Participants discussed several lifestyle factors they believed increased a person's risk for dementia. An unhealthy diet was discussed among several groups as a risk factor for dementia. The role of exercise or the lack of it was also seen by participants to modify risk for dementia. Having limited hobbies and interests, not partaking in social activities or being socially isolated were viewed by participants across all ethnic groups as increasing the risk for dementia. Some participants also raised the effects of smoking and substance misuse, namely alcohol and drugs, may also increase the likelihood of developing dementia.

Physical trauma

Some participants also raised that stroke, brain injury and playing contact sports may also increase the likelihood of developing dementia. When talking about contact sports one

participant spoke about football and how heading the ball may have contributed to some footballers developing dementia later in life.

Psychological Trauma

Participants also discussed how traumatic life events and stress may be linked to dementia. Some participants discussed how being anxious, overthinking or worrying may cause dementia. Other participants raised that depression may also be linked to getting dementia.

Environment

Participant discussed the role of environment, namely effects of pollution, socioeconomic disadvantage and varied access to good quality healthcare services. Participants felt that pollution may also increase dementia risk and queried whether there was any research happening around this. Also, some focus groups highlighted the impact of socioeconomic disadvantage and how that may increase dementia and impact what people have access to in terms of support and services. Participants also discussed at length the varied access to dementia specific services and healthcare services more generally. Participants felt that this inconsistent access may be linked to dementia in some way.

Existing conditions

Participants discussed whether having existing conditions such as hypertension, diabetes or depression increased the risk for dementia.

Faith / Spirituality

Across some of the focus groups, where participants were from Arab, South Asian and African communities and were also aligned with a particular faith or spiritual practice, faith and spirituality were presented as a double-edged sword with the potential to mitigate or worsen dementia depending on patients' choices. Some participants felt that those who did not engage with their faith, did not pray or engage with any sort of spiritual practice may be at increased risk of dementia. One participant who was Muslim talked about how the action of praying and bowing down with the head to the floor is also a form of exercise which may help protect against dementia. Other participants discussed how engaging with their faith, prayers and spiritual practice may help protect against dementia. Conversely, some participants felt prioritising religious and spiritual intervention over clinical can worsen dementia.

Level of Education

The level of education was also viewed as a risk factor for dementia by some participants.

Lack of sleep

Across many of the focus groups participants identified lack of sleep or problems with sleep as a risk factor for dementia. Participants discussed how sleep changes with age, with some participants reflecting on how their sleep pattern has changed as they have gotten older and how they struggle to sleep. They viewed challenges with sleeping as increasing one's risk for dementia.

Age

Participants associated old age with dementia and how older people were more likely to have dementia.

Hearing loss

One focus group discussed the impact of hearing loss and how this may be linked to an increased risk of dementia.
