# Supplementary material for: Qualitative study exploring knowledge and attitudes towards dementia risk prediction, barriers to dementia services and service improvement recommendations with diverse populations in England
Source: BMJ Open. 2025 May 30;15(5):e092370. doi: 10.1136/bmjopen-2024-092370 (PMC12128399; doi:10.1136/bmjopen-2024-092370)
Supplement: Supplementary file 5 [file bmjopen-2024-092370.reviewer_comments.pdf]

PEER REVIEW HISTORY

BMJ Open publishes all reviews undertaken for accepted manuscripts. Reviewers are asked to complete a checklist review form and are provided with free text boxes to elaborate on their assessment. These free text comments are reproduced below.

ARTICLE DETAILS

Title (Provisional)

A qualitative study exploring knowledge and attitudes towards dementia risk prediction, barriers to dementia services and service improvements recommendations with diverse populations in England

Authors

Bajwa, Rupinder; Hanjari, Matilda; Al-Oraibi, Amani; Akeya, Ralph; Brar, M; Robinson, Louise; Stephan, Blossom CM; Qureshi, Nadeem; Bains, Manpreet

---

VERSION 1 - REVIEW

**Reviewer**	**1**
**Name**	**Wang, Tianhao**
**Affiliation**	**Rush Alzheimer's Disease Center**
**Date**	**13-Oct-2024**
**COI**	**None**

This manuscript investigates several aspects of dementia awareness and services among diverse populations in England. Below are my major comments.

1. Please provide a complete distribution of the ethnic backgrounds of the participants (percentage adding to 100%) in the Abstract.

2. I am not sure whether a sample of 157 can be claimed to be "a large sample of participants", especially in the context of diverse communities where enough samples are required for each diverse community. Many ethic groups in this sample only had one digit sample size, which is a limitation rather than strength of this study.

3. The flow of the introduction is not very smooth. It first pointed out that reliable prediction tools for dementia was still lacking, and then proceeded that this issue was even more severe for underrepresented diverse groups. This is undoubtedly true. But it is unclear how this fact motivates the need to understand how the underserved groups perceive dementia and how much they can access dementia services, because comprehensive knowledge about dementia and reliable risk assessment are not available in reality anyway.

4. It seems the two sites recruited participants of different backgrounds. How to delineate the effects of different sites from the effects of different backgrounds?

**Reviewer**	**2**
**Name**	**Page, Amy**
**Affiliation**	**The University of Western Australia, Pharmacy Department**
**Date**	**18-Jan-2025**
**COI**	**None**

Thank you for the opportunity to review this paper. This manuscript presents a qualitative study exploring the knowledge and attitudes towards dementia risk prediction, barriers to accessing dementia services, and recommendations for service improvements among diverse populations in the UK. The study involved 157 participants from low-income and ethnically diverse backgrounds, using task group methodology and framework analysis. I have suggestions for its improvement that I hope are taken in the spirit they're intended.

Strengths and limitations of the study:

These could be more thoughtful. At the moment, they're just repeating the population more or less. Only the second dot point (We engaged in meaningful and reciprocal community engagement practices to enhance researcher-community relationships) is meaningful. Can you rewrite the others thinking of the strengths and weaknesses of your study that aren't just describing who your population is?

Introduction

The introduction is well written but may need some more nuanced discussion of the background. You state that the research currently is just limited to data from Caucasian populations, but then go on to discuss socioeconomic and include people who are Caucasian and from low socio economic backgrounds. Please just make the argument hang together a little more. I think it's an important point, but you just need to make sure that you're seperating out ethnicity and socio-economic factors in the introduction so that the story is striaght.

Overall:

Very well written and interesting study.

Please just revise the occassional language (e.g. limited knowledge prefered to lack of knowledge)

**Reviewer**	**3**

**Name**	**Bray, Jennifer**
**Affiliation**	**University of Worcester**
**Date**	**27-Jan-2025**
**COI**	**None**

It might look like a lot, but hopefully the comments below are all fairly minor and easy to address.

Line 125 – As you're working with groups that may not traditionally be involved in research and may have language barriers, I would be interested to know about the ethics process to know how people gave consent to be involved. Were there any issues, particularly if you had information sheets in English? Was consent written or given verbally? I would expect more information than just 'Patient consent: Obtained' in line 450. This is potentially a big learning point that could help others, but instead you've glossed over it.

Line 156 – You use PPI but don't say what it means.

Line 184 – You talk about field notes being written 'post-interviews' but your methodology doesn't use interviews, it uses focus groups. This inconsistency needs to be addressed to avoid confusion.

Line 188 – You should be consistent with your numbers. It should be nine and four not 9 and 4. You use six and two in the previous sentence, so it seems odd to switch to figures here.

Lines 189-190 – I would argue that your statement about gender is incorrect. By saying 'Among those who provided their gender' it implies that you're calculating your figures excluding the 20 participants who did not provide a gender, but that's not the case. Your 58% being female is based on the whole group of participants. I would suggest reworking the sentence to either say that 58% of participants were female, or 48% of participants who provided their gender were female.

Lines 190-192 – I find your statistics about ethnicities very confusing (I also wondered about it in the abstract, but thought I'd wait and see what it was like in the main article). My initial confusion was that the % given add up to nowhere near 100%, so what happened to the rest? I then thought you must just be mentioning the top few ethnicities, even though it seems odd to give 10.2% before 13.4% - I would have expected them to be in descending order. However, having looked at Table 1, I'm even more confused. If you were giving the top few ethnicities it should be British White (17.8%), Arab (13.4%), African (11.5%), British Asian (10.8%), British Asian – Pakistani (10.2%). What was the reasoning behind the list you give in the text? Unless there was a deliberate choice that can be explained, I would suggest something more logical.

By only listing four ethnicities then saying ‘0.6% from other ethnicities’ implies that everyone else fell into this group, which is clearly not the case. Table 1 shows that this is incorrect, as it’s not even ‘other ethnicities’, it’s ‘0.6% mixed’.

(Also, in Table 1, you are missing a ‘)’ after the not provided % of 36.3%)

Lines 200-202 – This sentence is odd, and I think it’s because of the word ‘including’. It makes the reader think that you are listing some of the sub-themes (as the list follows mention of them), but actually you are listing the overarching themes. As there are four of these, your list of four is not ‘including’, it is the whole list. I would suggest changing it to something like ‘Four overarching themes were generated, with corresponding sub-themes, as shown in Table 2. The results are presented under these themes and sub-themes.’ For me, this would let the reader know how the results are going to be shown, and remove the need to list the themes in the text as they are used as headings.

It would be useful if the sub-themes shown in Table 2 matched the sub-headings used in the results. For example, the text has a sub-heading ‘Knowledge about dementia, the symptoms and risk factors’ but Table 2 says ‘Knowledge symptoms and risk factors’. Some consistency would be good. I haven’t checked them all (although I have spotted that you use both ‘health care’ and ‘healthcare’), so it would be worth someone doing this.

Lines 214-215 – To avoid repetition, I would say ‘These risk factors included’ instead of ‘Participants discussed a range of risk factors including’.

Lines 234-236 – Are these two sentences needed? You’ve already shown the sub-themes in Table 2, and didn’t list them in the text for the first overarching them, so why list them here? Also, were attitudes really positive? There was no indication of attitude in the first sub-theme, and the second sub-theme seems to show quite negative attitudes. Only the final sub-theme seems to be positive, so I’m not sure that a statement implying that attitudes were positive overall is particularly accurate.

Line 262 – I would suggest saying ‘appointments, and limited consultation times not giving participants’ instead of ‘appointments and limited consultation times did not give participants’.

Lines 273-276 – These two sentences are quite repetitive. A suggested reworking could be to say ‘All participants felt that there was still a stigma attached to a dementia diagnosis, particularly within ethnic minority communities, and that this may hinder some from accessing the appropriate health services.’

Line 286 – It might read better if you say ‘memory, so they’ instead of ‘memory, they’.

Lines 291-292 – Instead of ‘Participants from these groups’ I think you could get away with just saying ‘They’.

Lines 328-331 – Similar to an earlier comment, I’m not sure it these sentences are needed.

Line 336 – A really minor point, but I think it should be ‘groups: (i) Cultural’ instead of ‘groups. (i) cultural’.

Line 375 – As per earlier email, I’m not convinced attitudes were positive.

VERSION 1 - AUTHOR RESPONSE

**Reviewer comments**	**Author’s response**
**Reviewer 1:**<br>**(Dr. Tianhao Wang, Rush Alzheimer’s Disease Center, Rush University Medical Center)**	
1. Please provide a complete distribution of the ethnic backgrounds of the participants (percentage adding to 100%) in the Abstract.	• This has been included and ethnicity categories used have been updated to improve readability.<br>• *‘Participants were from diverse ethnic backgrounds, with 35% Black or Black British, 23.6% Asian or Asian British, 18.5% White, 14.6% not specified, 7%, Arab, and 1.3% other ethnicities.’*
2. I am not sure whether a sample of 157 can be claimed to be “a large sample of participants”, especially in the context of diverse communities where enough samples are required for each diverse community. Many ethic groups in this sample only had one digit sample size, which is a limitation rather than strength of this study.	• Recruiting people from ethnic minority groups who are often marginalised, experience disparities in access and health outcomes, are often under-represented in health research. Evidence also shows that engaging with under-represented communities can be challenging. A key strength of the present study is that we were able to connect and engage with diverse communities and recruit a large sample of participants representing different socioeconomic and ethnic minority groups.<br>• However we do acknowledge that some of these groups are better represented in our sample than others. Despite the small numbers for some groups, the key strength of this work is that we were able to engage with these groups and they participated in this study.<br>• We have included the following in the strengths and limitations sections of the manuscript:

	• *'In this study, we engaged with diverse communities and recruited a large sample of participants representing low SES and ethnic minority groups. However, we do acknowledge that some of these groups are better represented in our sample than others. Despite the small numbers for some groups, the key strength of this work is that we were able to engage with these groups and they participated in the study.'*
3. The flow of the introduction is not very smooth. It first pointed out that reliable prediction tools for dementia was still lacking, and then proceeded that this issue was even more severe for underrepresented diverse groups. This is undoubtedly true. But it is unclear how this fact motivates the need to understand how the underserved groups perceive dementia and how much they can access dementia services, because comprehensive knowledge about dementia and reliable risk assessment are not available in reality anyway.	• This has been edited to improve the flow. Please refer to the manuscript.
4. It seems the two sites recruited participants of different backgrounds. How to delineate the effects of different sites from the effects of different backgrounds?	• Preliminary analysis looked at within and between group differences (site, SES and ethnicity). No site specific differences in themes were found. Ethnicity-specific findings were reported across several themes including knowledge about dementia, symptoms and risk factors, fear and stigma, denial, cultural and religious beliefs about dementia and cultural approaches to caring for elders.<br>• In the results section under the 'Thematic Framework' heading we have included the following text:<br><br>• *'The preliminary analysis looked at within and between-group differences. Findings were consistent across the sites, however, some ethnicity-related differences were*

<table>
<tr><td></td><td>noted and have been reported within the corresponding themes.’</td></tr>
<tr><td colspan="2">Reviewer 2:
(Dr. Amy Page, The University of Western Australia, Monash University)</td></tr>
<tr><td>5. Introduction
The introduction is well written but may need some more nuanced discussion of the background. You state that the research currently is just limited to data from Caucasian populations, but then go on to discuss socioeconomic and include people who are Caucasian and from low socio economic backgrounds. Please just make the argument hang together a little more. I think it's an important point, but you just need to make sure that you're separating out ethnicity and socio-economic factors in the introduction so that the story is straight.</td><td>• The introduction has been edited to clarify the issue around dementia prediction models being developed using data from affluent Caucasian populations. Thus we wanted to examine experiences from low income White groups.</td></tr>
<tr><td>6. Strengths and limitations of the study: These could be more thoughtful. At the moment, they're just repeating the population more or less. Only the second dot point (We engaged in meaningful and reciprocal community engagement practices to enhance researcher-community relationships) is meaningful. Can you rewrite the others thinking of the strengths and weaknesses of your study that aren't just describing who your population is?</td><td>The strengths and limitations section has been updated to include the following:

• ‘We considered the needs and cultural norms of the communities we engaged with by running single-gender focus group sessions where this was preferred and providing language support via translation of materials and interpretation during focus groups.’

• ‘We found no site-related differences in our findings, which enhances the transferability of the findings to diverse groups in different regions of the UK’.</td></tr>
<tr><td>7. Please just revise the occasional language (e.g. limited knowledge prefered to lack of knowledge)</td><td>• Phrasing has been revised in the manuscript.</td></tr>
<tr><td colspan="2">Reviewer 3:
Ms. Jennifer Bray, University of Worcester</td></tr>
<tr><td>8. Line 125 – As you’re working with groups that may not traditionally be</td><td>• We have updated the methods section to provide more detail about</td></tr>
</table>

involved in research and may have language barriers, I would be interested to know about the ethics process to know how people gave consent to be involved. Were there any issues, particularly if you had information sheets in English? Was consent written or given verbally? I would expect more information than just 'Patient consent: Obtained' in line 450. This is potentially a big learning point that could help others, but instead you've glossed over it.	the process for consent and how we account for language barriers. Please see heading 'Informed Consent':<br>• *'All participants were provided with a Participant Information Sheet via e-mail or in person (via gatekeeper or attending local community settings), ensuring that the participant had*<br>• *sufficient time to consider participation. Gatekeepers helped interpret for members who had English language difficulties to aid understanding. All participants provided written informed consent, completing either a digital copy or paper copy of the consent form, before participating in a focus group.'*
9. Line 156 – You use PPI but don't say what it means.	• Please see addition of the 'Patient and Public Involvement' heading in methods section:<br>• *'We engaged with existing PPI representatives (from the Dementia Care Community Group in Newcastle) and local community gatekeepers at both sites to help design, refine and pilot approaches to recruitment, focus group delivery and the content to be delivered using task group methodology. We also sought input from these stakeholders on topic guide development to ensure that the language was appropriate, and questions were clear. Public engagement enabled the research team to understand different cultural norms and practices across communities and how to consider this to encourage participation and ensure approaches were culturally sensitive and inclusive.'*
10. Line 178 – You talk about field notes being written 'post-interviews' but your methodology doesn't use interviews, it uses focus groups. This inconsistency needs to be addressed to avoid confusion.	• This has been corrected to ensure consistency with the methodology used.
11. Line 184 – You should be consistent with your numbers. It should be nine and four not 9 and 4. You use six and	• This has been amended to all numerical figures in the text to ensure consistency.

two in the previous sentence, so it seems odd to switch to figures here.	
12. Lines 189-190 – I would argue that your statement about gender is incorrect. By saying 'Among those who provided their gender' it implies that you're calculating your figures excluding the 20 participants who did not provide a gender, but that's not the case. Your 58% being female is based on the whole group of participants. I would suggest reworking the sentence to either say that 58% of participants were female, or 48% of participants who provided their gender were female.	• This has been updated to improve clarity.<br>• *'Fifty-eight per cent of participants were female (n=91).'*
13. Lines 190-192 – I find your statistics about ethnicities very confusing (I also wondered about it in the abstract, but thought I'd wait and see what it was like in the main article). My initial confusion was that the % given add up to nowhere near 100%, so what happened to the rest? I then thought you must just be mentioning the top few ethnicities, even though it seems odd to give 10.2% before 13.4% - I would have expected them to be in descending order. However, having looked at Table 1, I'm even more confused. If you were giving the top few ethnicities it should be British White (17.8%), Arab (13.4%), African (11.5%), British Asian (10.8%), British Asian – Pakistani (10.2%). What was the reasoning behind the list you give in the text? Unless there was a deliberate choice that can be explained, I would suggest something more logical.<br><br>By only listing four ethnicities then saying '0.6% from other ethnicities' implies that everyone else fell into this group, which is clearly not the case. Table 1 shows that this is incorrect, as it's not even 'other ethnicities', it's '0.6% mixed'.<br><br>(Also, in Table 1, you are missing a ')' after the not provided % of 36.3%)	• Thank you for highlighting this, we agree that this is quite confusing to read. We have edited table 1 to improve the readability of the data.

14. Lines 195-197 – This sentence is odd, and I think it's because of the word 'including'. It makes the reader think that you are listing some of the sub-themes (as the list follows mention of them), but actually you are listing the overarching themes. As there are four of these, your list of four is not 'including', it is the whole list. I would suggest changing it to something like 'Four overarching themes were generated, with corresponding sub-themes, as shown in Table 2. The results are presented under these themes and sub-themes.' For me, this would let the reader know how the results are going to be shown, and remove the need to list the themes in the text as they are used as headings.<br><br>It would be useful if the sub-themes shown in Table 2 matched the sub-headings used in the results. For example, the text has a sub-heading 'Knowledge about dementia, the symptoms and risk factors' but Table 2 says 'Knowledge symptoms and risk factors'. Some consistency would be good. I haven't checked them all (although I have spotted that you use both 'health care' and 'healthcare'), so it would be worth someone doing this.	• The sentence has been reworded as suggested to improve clarity.<br>• '*Four overarching themes were generated, with corresponding sub-themes as shown in Table 2. The results are presented under these themes and sub-themes.'*<br><br>• Table 2 and sub-theme headings have been corrected to ensure consistency.
15. Lines 208-209 – To avoid repetition, I would say 'These risk factors included' instead of 'Participants discussed a range of risk factors including'.	• This has been edited to remove the repetition.
16. Lines 228-230 – Are these two sentences needed? You've already shown the sub-themes in Table 2, and didn't list them in the text for the first overarching them, so why list them here? Also, were attitudes really positive? There was no indication of attitude in the first sub-theme, and the second sub-theme seems to show quite negative attitudes. Only the final sub-theme seems to be positive, so I'm not sure that a statement implying that attitudes were positive overall is particularly accurate.	• These sentences have been removed to improve clarity.

17. Line 252 – I would suggest saying 'appointments, and limited consultation times not giving participants' instead of 'appointments and limited consultation times did not give participants'.	• This has been amended to improve clarity<br>• '*For primary care services, challenges included not being able to book an appointment with a general practitioner (GP), a lack of continuity with GPs, including not seeing the same one for subsequent appointments and limited consultation times not giving participants sufficient time to raise the issues they wanted to address with their GP.'*
18. Lines 263-266 – These two sentences are quite repetitive. A suggested reworking could be to say 'All participants felt that there was still a stigma attached to a dementia diagnosis, particularly within ethnic minority communities, and that this may hinder some from accessing the appropriate health services.'	• This has been edited to reduce repetition.<br>• *'All participants felt that there was still a stigma attached to a dementia diagnosis, particularly within ethnic minority communities, and that this may hinder some from accessing the appropriate health services.'*
19. Line 286 – It might read better if you say 'memory, so they' instead of 'memory, they'.	• This has been amended to improve readability.<br>• *'Participants from African communities highlighted how Black men are proud and may struggle to admit they may have problems with their memory, so they may choose to suffer in silence instead of seeking support.'*
20. Lines 280-281 – Instead of 'Participants from these groups' I think you could get away with just saying 'They'.	• This has been amended to be more succinct as suggested by the reviewer.<br>• *'They highlighted how people within these communities viewed memory loss as common in later life and "it happens to everyone."'*
21. Lines 317-320 – Similar to an earlier comment, I'm not sure it these sentences are needed.	• The sentences have been removed.
22. Line 336 – A really minor point, but I think it should be 'groups: (i) Cultural' instead of 'groups. (i) cultural'.	• Thank you for highlighting this, this has now been corrected.
23. Line 375 – As per earlier email, I'm not convinced attitudes were positive.	• After discussions with the team, we have updated the phrasing to '*cautiously optimistic*'. We feel this better reflects attitudes towards dementia risk prediction tools. Participants saw the potential

	benefits but were cautious, highlighting the need for advice to support with making changes to lower risk. Participants were also concerned about the psychological impacts of a high risk result.

VERSION 2 - REVIEW

**Reviewer**	**3**
**Name**	**Bray, Jennifer**
**Affiliation**	**University of Worcester**
**Date**	**21-Mar-2025**
**COI**	

Thank you for the work you have done to address previous review comments. I don't have comments really about the content, but think there are a couple of minor areas to address.

Line 163 – sorry, I wasn't clear in my previous round of comments. When I said about PPI, I didn't mean that you didn't say what you meant by PPI, I meant that you hadn't set up the acronym/abbreviation. For example, in line 87 you said 'socioeconomic status (SES)', so you need to say 'Patient and Public Involvement (PPI)' here.

Line 175 – you talk about audio recordings, but haven't previously said that the focus groups were audio recorded. It is probably worth mentioning this somewhere in your Data collection section.

Lines 199-200 – I think it's generally the case that numbers up to and including ten are written as words rather than figures, so it might be worth checking these two lines.

Table 1 – I think this would benefit from a bit of formatting to ensure consistency (font size) and making it easier to read/follow. Currently, the ethnicity heading does not align with the first subgroup, which could potentially cause confusion.

In line 245 you give the second overarching theme before going onto the sub-themes, but you didn't have a heading for the first overarching theme. Some consistency would be good.

Line 265 – the overarching theme doesn't quite match what you say in Table 2. It would be good to have consistency.

Line 266 – you say 'health care' here but 'healthcare' in Table 2. Which is it? Check what you use throughout the article for consistency.

Line 297 – you say 'aging' here but 'ageing' in Table 2. Which is it? I assume 'aging' as that's what you use in lines 299 and 301 as well, but it is worth checking what you use throughout the article for consistency.

I'm not going to go through each sub-theme as you should get the idea, but there are other ones where the heading doesn't match Table 2. I know it's really picky, but having things match and be consistent feels like a really simple thing to check and fix – it's frustrating as I know I raised it in my previous round of comments and in your response document you said 'Table 2 and sub-theme headings have been corrected to ensure consistency' which is clearly not the case.

Line 430 – why do you mention single-gender focus groups here when you didn't say anything about this in your data collection section? It feels odd to raise a new point here. You say about using different languages in the focus groups in line 147, so why not also say about some being single-gender as well?

VERSION 2 - AUTHOR RESPONSE

**Reviewer 3:**<br>**Ms. Jennifer Bray, University of Worcester**	
1. Line 163 – sorry, I wasn't clear in my previous round of comments. When I said about PPI, I didn't mean that you didn't say what you meant by PPI, I meant that you hadn't set up the acronym/abbreviation. For example, in line 87 you said 'socioeconomic status (SES)', so you need to say 'Patient and Public Involvement (PPI)' here.<br>a.	• The abbreviation has now been set up accordingly.
2. Line 175 – you talk about audio recordings, but haven't previously said that the focus groups were audio recorded. It is probably worth mentioning this somewhere in your Data collection section.	• We have now mentioned that audio recording of sessions in the data collection section.<br>• '*Sessions were audio recorded*'.
3. Lines 199-200 – I think it's generally the case that numbers up to and including ten are written as words rather than figures, so it might be worth checking these two lines.	• Lines 199-200 numbers under 10 have been written as words.

4. Table 1 – I think this would benefit from a bit of formatting to ensure consistency (font size) and making it easier to read/follow. Currently, the ethnicity heading does not align with the first subgroup, which could potentially cause confusion.	• The table has been formatted for consistency across font, font size to make it easier to follow. Heading alignment has also been amended.
5. In line 245 you give the second overarching theme before going onto the sub-themes, but you didn't have a heading for the first overarching theme. Some consistency would be good.	• For consistency we have now included the heading for the first overarching theme.
6. Line 265 – the overarching theme doesn't quite match what you say in Table 2. It would be good to have consistency.	• This has now been updated to ensure consistency between the heading and table 2.
7. Line 266 – you say 'health care' here but 'healthcare' in Table 2. Which is it? Check what you use throughout the article for consistency.	• This has now been updated for consistency and 'health care' has been changed to 'healthcare' throughout the manuscript.
8. Line 297 – you say 'aging' here but 'ageing' in Table 2. Which is it? I assume 'aging' as that's what you use in lines 299 and 301 as well, but it is worth checking what you use throughout the article for consistency.	• In line with British English standard, 'Aging' has been replaced with 'Ageing' throughout the document,
9. I'm not going to go through each sub-theme as you should get the idea, but there are other ones where the heading doesn't match Table 2. I know it's really picky, but having things match and be consistent feels like a really simple thing to check and fix – it's frustrating as I know I raised it in my previous round of comments and in your response document you said 'Table 2 and sub-theme headings have been corrected to ensure consistency' which is clearly not the case.	• The headings in text and table 2 have been corrected to ensure consistency throughout the manuscript.
10. Line 430 – why do you mention single-gender focus groups here when you didn't say anything about this in your data collection section? It feels odd to raise a new point here. You say about using different languages in the focus	• Line 147, we have now included that sessions were single-gender sessions in line with communities' preferences. • '*Where feasible, specific groups were run in languages other than English or*

groups in line 147, so why not also say about some being single-gender as well?	*were single gender sessions in line with communities' preferences'*
